# The Analysis of Additive Manufacturing Parameters to Evaluate the Feasibility of Using Spare Parts in the Shipbuilding Industry

**DOI:** 10.3390/ma19214631

**Published:** 2026-10-31

**Authors:** Tomasz Cyryl Dyl

**Affiliations:** Department of Marine Maintenance, Faculty of Marine Engineering, Gdynia Maritime University, Morska Street 81–87, 81-225 Gdynia, Poland; t.dyl@wm.umg.edu.pl

**Keywords:** additive manufacturing, technological parameters, spare parts, Industry 4.0, shipbuilding industry

## Abstract

This article discusses a modern manufacturing method: additive manufacturing technology. It outlines the development trends of production methods in the shipbuilding industry, forecasts for the coming years, and the factors driving the development and implementation of new manufacturing technologies while also considering the historical context. Furthermore, the article characterizes additive methods in line with the current literature and highlights the features and advantages of using these technologies to produce parts and components for marine machinery. The rapid development of additive manufacturing technology in recent years and its promising future have prompted certain classification societies to develop publications containing guidelines and recommendations for additive manufacturing methods, the application of which enables the certification of final products. Several case studies involving machinery components such as a ship propeller and a pressure pipe are described. Additionally, a roadmap for the Shipyard 4.0 concept is included.

## 1. Introduction

Additive manufacturing (AM) is undoubtedly one of the modern production methods, representing a breakthrough in how final products are shaped. This technology aligns with the Industry 4.0 concept and has the potential to transform the industry toward highly efficient, and production-ready component production, characterized by a high degree of process computerization.

In addition to cost savings and waste reduction, additive manufacturing enables rapid prototyping, immediate design changes, and the production of components with highly complex geometries that are impossible to achieve using conventional methods.

Nowadays, rapid prototyping and rapid tooling methods are exerting an increasingly profound influence on manufacturing processes. A growing number of companies in the 3D printing sector are gradually expanding their operations to include rapid manufacturing services, that is, the direct production of end-use products and tooling. These components possess all the properties of market-ready products. However, the technology still faces numerous challenges, such as limited production speeds, the range of available materials, and manufacturing accuracy. Nevertheless, given the increasing number of successful applications, it is anticipated that many of these obstacles will be overcome in the coming years and that this type of end-part manufacturing technology represents the future of 3D printing.

**Figure 1:**
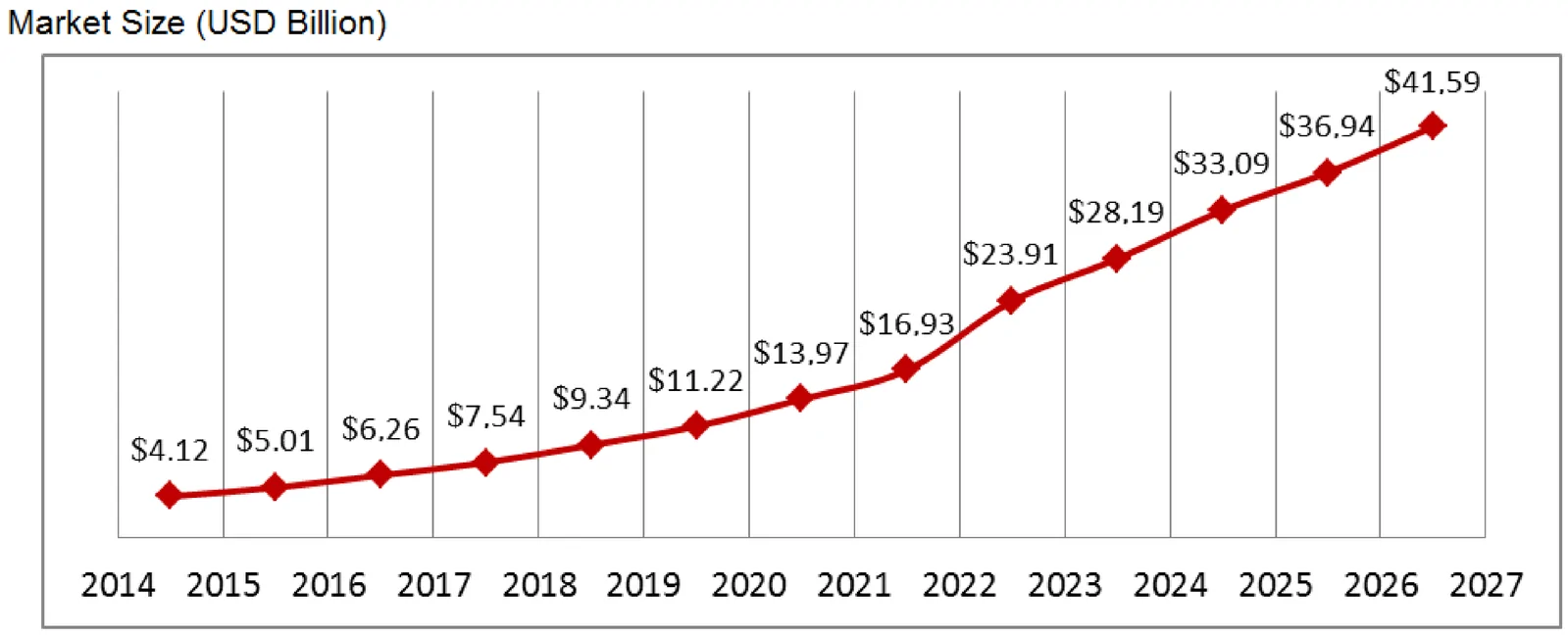
The value of the additive manufacturing industry in the coming years [1].

According to SmartTech Publishing, a leading analytical company specializing in the additive manufacturing market, the 3D printing industry is estimated to be worth over USD 4.12 billion (in 2014), and forecasts indicate that by 2027 its value will exceed USD 41.59 billion (Figure 1) [1]. Driven by rapid advancements including reduced production costs and improvements in material properties, part quality, and precision, 3D printing is regarded as a cost-effective solution for manufacturing demanding, complex industrial products. The aerospace, space, automotive and shipbuilding industry sectors are among the primary industries adopting this technology for the production and refinement of machinery, equipment, components, and other structural elements.

AM has enormous potential in the repair of ship parts, both on land and at sea. Unfortunately, there is no single universal 3D printing method suitable for the production or repair of all components with equally good results. Equipping a shore-based facility with several printing devices using different technologies is not a problem, but onboard this is not possible due to limited space and the expected low return on investment if multiple devices are purchased [1,2].

To implement modern manufacturing methods on an industrial scale, it is necessary to first guarantee the appropriate quality and safety of products manufactured using these methods. To this end, it is necessary to ensure industry-specific regulations that would allow for the implementation of certification of the manufacturing process and the final product for the technology used to produce this component, thus confirming the product’s suitability for its intended use. The intensive development of additive manufacturing technology recorded over the last few years and good forecasts for the coming years have contributed to the issuance by some classification societies of publications containing guidelines [1] and recommendations relating to additive production methods, the use of which will allow for obtaining certification of the final product. The development of modern manufacturing methods is driven primarily by the intense globalization of the market economy, which impacts product quality as well as the scale and speed of production.

As noted by Chlebus [3], key aspects of a competitive enterprise include order fulfillment time, production preparation time, adherence to schedules, the level of cooperation, and the ability to rapidly introduce new products and respond quickly to changes.

Cegielski et al. [4] point out that modern manufacturing methods (such as 3D printing) enable a significant reduction in the time required to develop and produce models, prototypes, and fully functional parts. According to a report published by Grand View Research [5], the global additive manufacturing (3D printing) market value is projected to grow from USD 37.6 billion in 2026 to USD 168.9 billion by 2033, representing a compound annual growth rate of 23.9% over the 2026–2033 period.

In 2025, the North American market held a dominant position, accounting for 32.8% of total revenue. Market growth is driven by the rapid adoption of industrial 3D printing, digital manufacturing methods, and Industry 4.0 technologies, as well as by increasing demand for on-demand production, mass customization, and rapid prototyping across sectors such as aerospace, healthcare, automotive, defense, and the shipbuilding industry. Currently, due to relatively low investment and material costs which ensure process cost-effectiveness, technologies involving the deposition of wire-based material into a geometric structure using a robotic arm and shielding gas are considered popular additive manufacturing methods.

The WAM^®^ and WAAM methods utilize technologies based on plasma and laser cladding processes; these were introduced by Klimpel [6] and described in articles by Bober [7] and Górka et al. [8], where the authors highlight the industrial applications of cladding technology. The widespread adoption of additive manufacturing methods is justified by the technology’s numerous advantages, including reduced production costs [9,10], shorter production times, lower product weight, the ability to consolidate parts, the capacity to create highly complex geometric structures [11,12], flexible production management [13], a reduced environmental footprint, and superior strength properties [14]. Issues regarding the profitability and viability of employing additive methods in production processes, including a cost model and forecasts for future years, were addressed in publication 1176 by the U.S. National Institute of Standards and Technology (NIST) [13].

The benefits of using additive manufacturing methods, along with potential applications for the technology in the maritime sector, have been outlined by Ziółkowski et al. [15]. The Green Ship of the Future organization, funded by the Danish Maritime Fund and collaborating with companies such as Alfa Laval, DNV GL, Man Energy Solutions, and Maersk, is conducting extensive research into the potential use of 3D printing in routine repairs. Between 2016 and 2018, they conducted trials implementing 3D printers on board ships [1,15]. They deployed nine ME printers on six ships and two platforms. Crews were to use 3D printing for routine repairs and to produce tools and instruments useful for daily operation and repairs. While precise results of the research are not available, the available data [1,15] suggests that crews did not fully utilize the delivered devices. This could be due to the quality and limitations of the entrusted equipment, the lack of training, the lack of design capabilities or skills, or the failure to provide appropriate 3D models.

Pragana et al. [16] presented a classification of additive methods into indirect and direct categories, along with a description of the processes associated with additive technology. As noted by Arrizubieta et al. [17], additive manufacturing technology is considered less harmful to the environment than conventional methods. Publications by classification societies regarding the specific technology employed can prove helpful in defining powder handling procedures throughout the product’s lifecycle. The guidelines issued by these societies also aim to establish procedures that ensure appropriate product quality and safety [18].

## 2. Materials and Methods

Although the level of 3D printing technology adoption in the maritime industry falls significantly short of that of the automotive or aviation industries, some shipping companies, shipbuilders, and port authorities have taken concrete steps to explore and accelerate its implementation. One of the main goals is to equip vessels with printing devices that would allow for spare parts to be printed onboard when needed, eliminating the need for lifeboats and helicopters. This would result in significant savings in time, money, and emissions.

Another aim is to equip shipyards with printing devices or create 3D printing centers in ports. Manufacturing products on demand anywhere would reduce the costs of storing and shipping spare parts to ships. Delays caused by onboard machinery failures could also be reduced, as spare parts would be manufactured at the next port rather than shipped from the central warehouse of the original equipment manufacturer. Essential small parts such as valves, pipe fittings or impellers can potentially be made on board in the event of a failure.

Ship operation is a complex process influenced by a large number of factors, events, and activities performed by the crew and external workers. Therefore, it is difficult to predict which components will fail, over what period, and under what conditions. In this case, we can only rely on historical data from the operation of similar vessels and manufacturer data, which can help us estimate the approximate time at which a given component will wear out. Onboard a ship, there is virtually constant rocking and vibration, and the temperature changes dramatically depending on the season, time of day, and cruising area. All of these factors can negatively impact print quality. Operating advanced 3D printers also requires appropriate training, a range of specialized materials, and conditions that ensure proper storage and operation. Vibration, humidity, seawater, and extreme temperatures can negatively impact the device and its materials. While these factors can be avoided by using appropriate materials and shipboard installations, they significantly increase implementation costs. At present, the only viable option seems to be the ME method using fiber-reinforced composites. Their very high mechanical properties allow for the fabrication of devices capable of withstanding high loads, while both the device and the materials are relatively inexpensive. This method can be used to produce special terminals, fasteners, clamps, tools, holders, washers, housings, covers, and other simple components that are not critical to the safety of navigation, as well as emergency parts that will last until the nearest port for replacement with a high-quality part.

**Figure 2:**
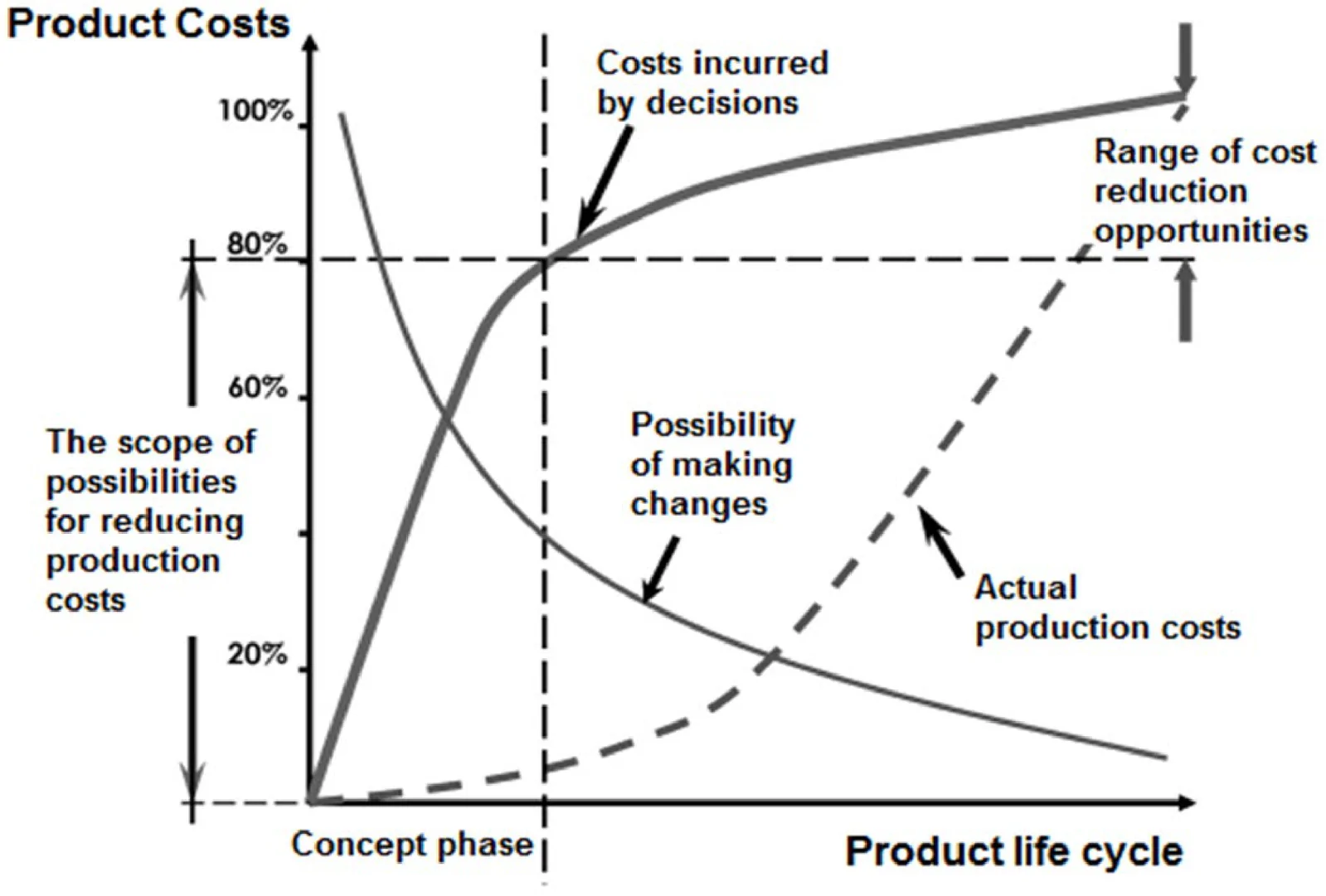
Possibilities of reducing production costs during the product life cycle [1].

**Figure 3:**
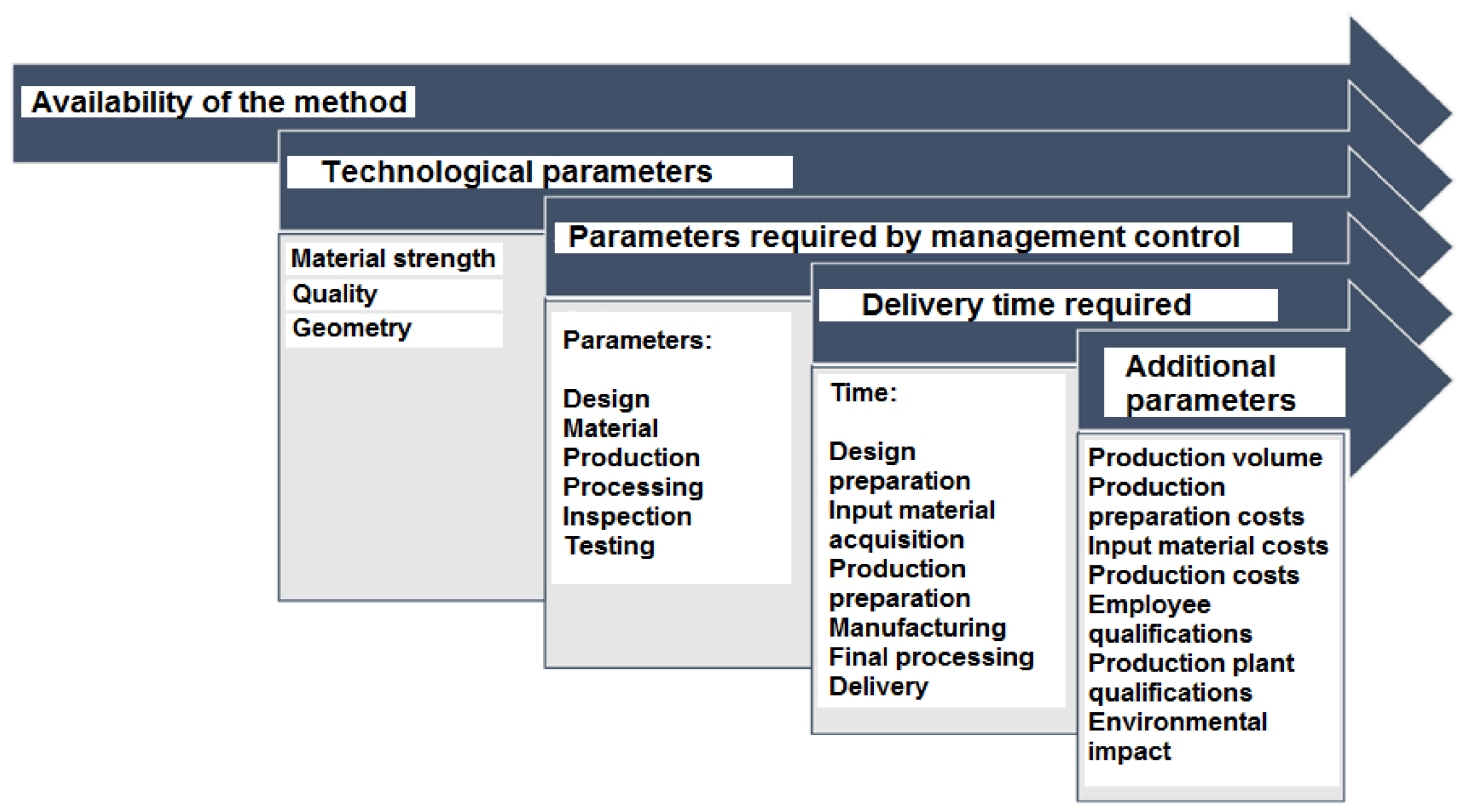
Diagram of the decision-making procedure in selecting a production method [1].

A properly conducted analysis should lead to the greatest possible reduction in production costs in subsequent stages. As shown in Figure 2, during the conceptual phase, the costs of subsequent production processes can be reduced by as much as 70–80%. The choice of manufacturing method depends on numerous factors, each of which should be considered at the production design stage. A diagram of the decision-making process for selecting a manufacturing method, along with key parameters influencing the final result, is shown in Figure 3.

The spectrum of 3D modeling techniques encompasses dozens of methods for forming objects directly from solid material, as well as from powder, liquid, and gas phases [19]. Typical general-purpose 3D printers produce models made of plastic; when it comes to machine components, the objects created in this way generally serve only for visualization purposes.

**Figure 4:**
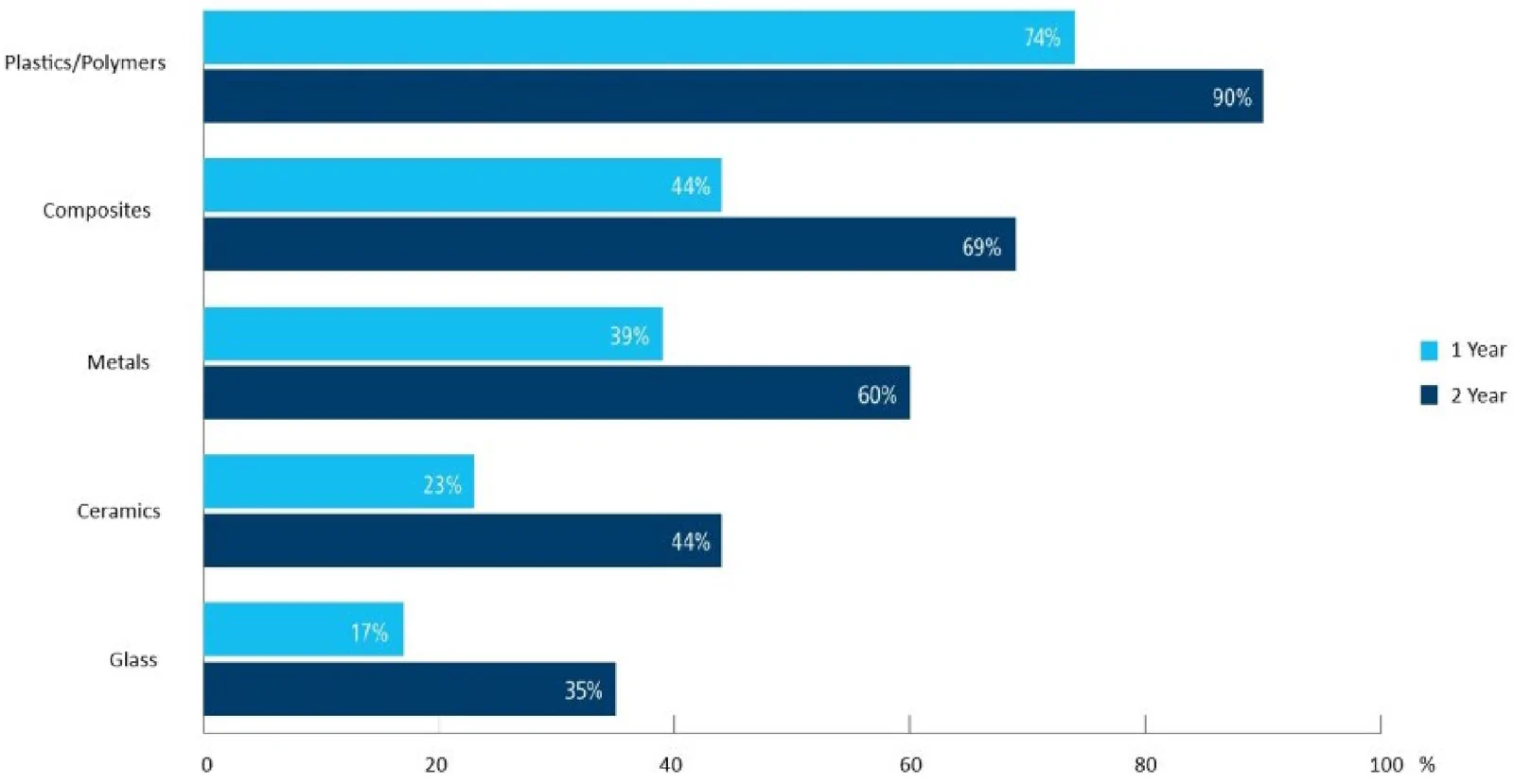
Materials used in additive manufacturing processes research conducted by the international company JABIL (1 Year—2019) and (2 Year—2021) [21].

Fully functional additives require specialized, advanced manufacturing methods that entail the highest costs. These include commercial-based methods, such as [19]: Material Extrusion (ME)—a thermoplastic material is extruded through a nozzle, where it is heated and then deposited layer by layer to create an object; Electron Beam Melting (EBM)—melting of metal powders using an electron beam; Selective Laser Sintering (SLS)—selective laser sintering of powders, most commonly plastics (polyamides) and metals; Laser Engineering Net Shaping (LENS) is a process for manufacturing components from powdered materials through localized deposition and laser beam sintering. In another case, regarding the SLS/SLM method, the powder is not distributed throughout the entire working chamber, but delivered to a specific target location; therefore, the LENS method also enables local repair (renewal) of existing machine parts. Selective Laser Melting (SLM) is the selective melting of powders using a laser beam; the lasers used in the SLM method must have higher power than those in SLS (in SLM, the powdered material undergoes complete melting, resulting in pore-free components). Laser Cladding (LC) is a combination of depositing powdered metal melted by a laser beam, after the application of each successive layer. Thermal surfacing is a method of 3D model fabrication. Wire Arc Additive Manufacturing (WAAM) technology utilizes MIG/MAG or TIG arc welding methods to manufacture objects additively; the material is fed in the form of wire, and the welding process takes place in a shielding gas atmosphere. 3D printing has revolutionized many industries, enabling the production of complex shapes with high precision and efficiency. As noted in [20], a wide range of 3D printing materials are used, such as metals, ceramics, thermoplastics, and bio-based materials. A key aspect of practical 3D component manufacturing is their characterization, which includes the assessment of mechanical properties and microstructural analysis. Above all, this AM technology can be applied in the aerospace, automotive, shipbuilding, medical, architectural, and fashion industries. By presenting the case study literature, it can be stated that research conducted by the international company JABIL [21] based on the experiences of 302 survey respondents representing key manufacturing companies across various industries with annual revenues exceeding USD 500 million shows a 21% increase in the share of metal materials used in additive manufacturing processes within the surveyed companies between 2019 (1 Year) and 2021 (2 Year). The survey results are presented in Figure 4.

Based on the case study literature, it can be concluded that the companies participating in the study clearly demonstrate a growing trend in the implementation of 3D printing in all business areas examined. Of particular interest is the significant growth in the use of additive methods for end product manufacturing, which rose by 35% between 2017 and 2021 [21]. Over a period of approximately two years, the use of all material types saw a sharp increase. Plastics and polymers remain at the top of the rankings, with 74% of respondents stating that their companies use plastics/polymers; composites now trail that figure by only 5%. Metals have made significant strides, growing by more than 20%. Several cases of components manufactured using modern manufacturing methods were considered: WAAM and ME.

**Figure 5:**
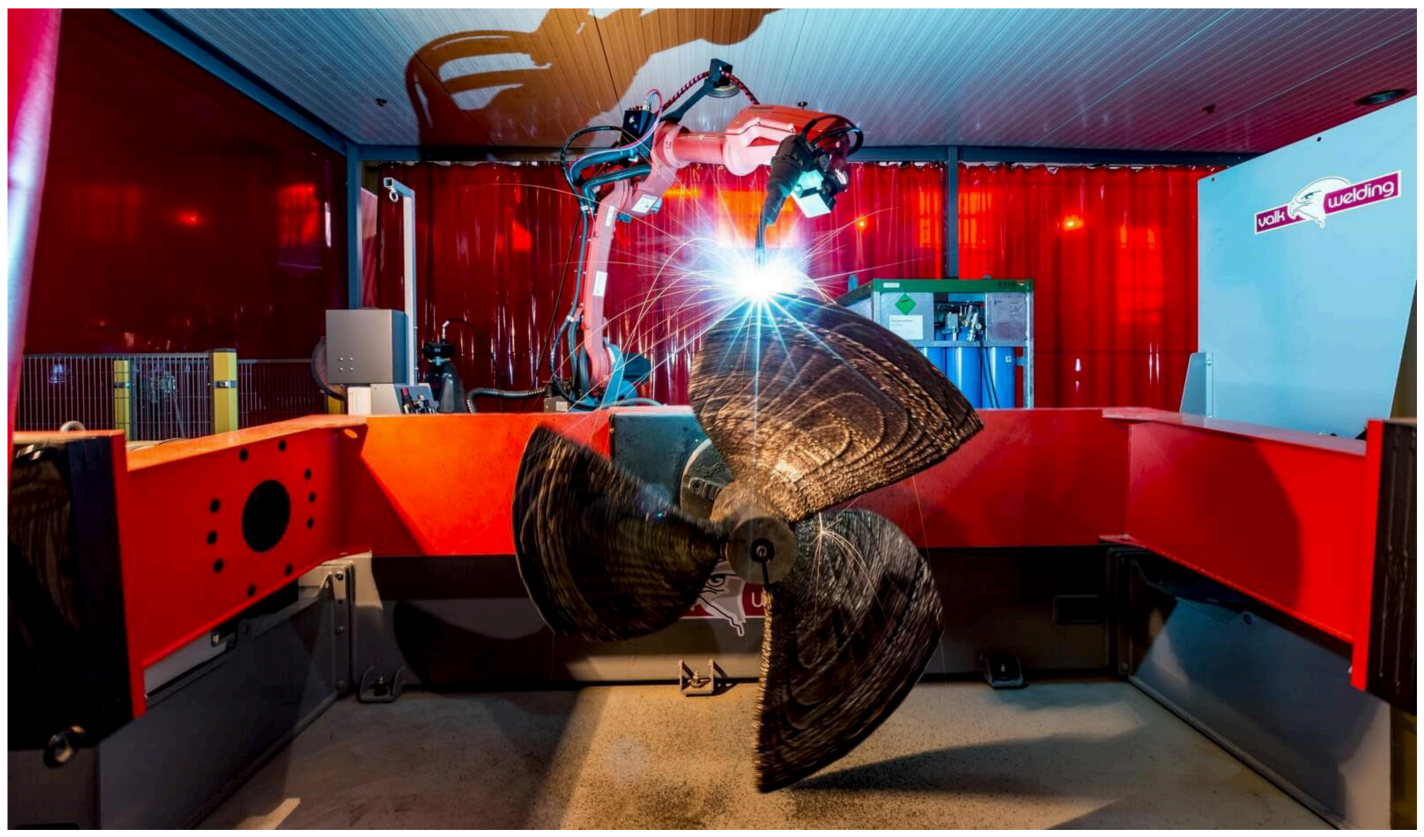
The manufacturing process of the WAAMpeller, example of a printed ship propeller, electric arc additive manufacturing [22,23].

In 2016, a pilot project was launched, spearheaded by Innovation Quarter, RDM Makerspace, and the Port of Rotterdam authorities, with the participation of dozens of other companies and agencies to explore the potential of using 3D printing to manufacture ship spare parts. Following a series of analyses and tests involving various additive manufacturing methods, it was determined that 3D printing technology could meet the established requirements in many instances. This pioneering collaboration resulted in the 2017 creation of the WAAMpeller, the world’s first 3D-printed ship propeller. The propeller was manufactured for and in cooperation with the DAMEN shipyard, which used it to power a Stan Tug 1606 tugboat (Figure 5). The propeller was developed at the Rotterdam Additive Manufacturing LAB, a research institute established specifically for this purpose at the Port of Rotterdam. The printing process employs Wire Arc Additive Manufacturing (WAAM) [22], a method in which material is fed as wire into an electric arc, melted, and deposited layer by layer to form the finished object. Another example of 3D printing technology implementation in the maritime sector is a pilot project launched in 2017 [15,23] by the Green Ship of the Future consortium. This consortium comprising over 20 industry partners works to enhance the sustainability of the maritime industry, including through the research of new technologies. The initiative is funded by the Danish Maritime Fund. The project involves supplying 3D printers to various locations including ships and drilling rigs along with training tools and videos, enabling crews to learn the 3D printing process and become self-sufficient in producing necessary parts. Additionally, in late 2018, the Maritime and Port Authority of Singapore signed an agreement with the leading global port group PSA, NAMIC, and the US-based 3D printing company 3D MetalForge to establish the world’s first additive manufacturing facility for port machinery components. Prior to this, the Maritime and Port Authority of Singapore had also collaborated with the renowned maritime industry advisor DNV GL and members of the Singapore Shipping Association to explore the potential of using 3D printing technology to manufacture spare parts for the maritime industry [23]. The entire manufacturing process was supervised by Bureau Veritas, which also issued certification for the finished propeller. During the production process, a set of samples was produced for tensile strength and maximum static load testing, while the finished propeller underwent sea trials; the test program included bollard pull and emergency stop tests in addition to speed trials. The testing culminated in obtaining classification society certification. RAMLAB continues to work on developing WAAM additive manufacturing methods as one of 21 partners from eight EU countries participating in the Grade2XL project [23,24].

In 2021, the Australian company AML3D, a global provider of large-scale metal additive manufacturing technologies and solutions, produced the world’s largest high-pressure pipeline component for the petrochemical industry using its proprietary WAM^®^ (Wire Additive Manufacturing) technology; the part was successfully tested and verified by Lloyd’s Register. This demonstrator component was manufactured as part of AML3D’s internal development program and showcases the potential for applying additive methods to production processes in the offshore petrochemical sector [10,25].

**Figure 6:**
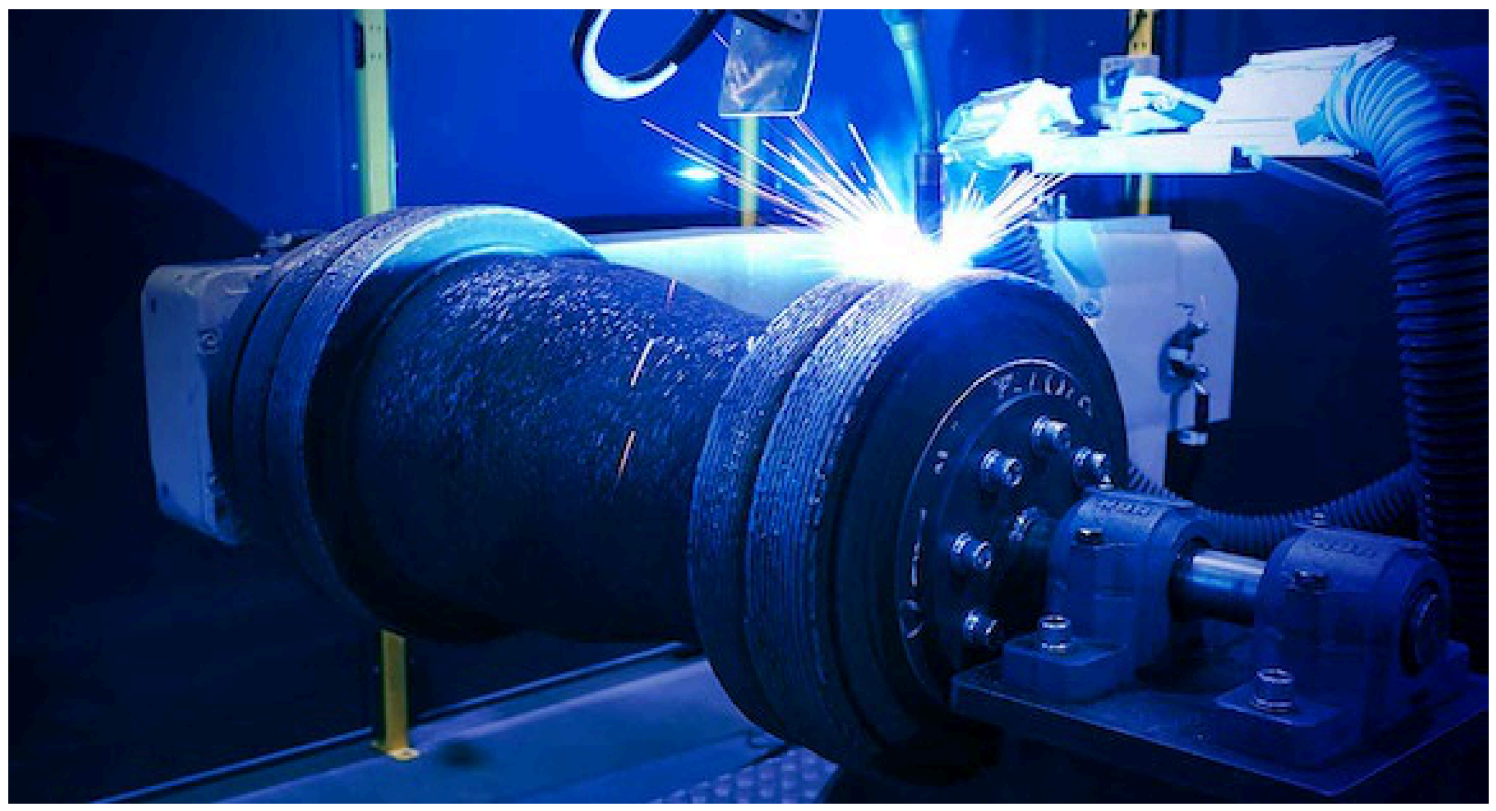
The Manufacturing Process of the Pipe DN400/DN300 Schedule 160 [10,25].

Weighing 940 kg, this reducer pipe (Figure 6) is the first component of its kind produced using metal additive manufacturing to undergo independent pressure testing globally. Measuring 850 mm in length, 450 mm in diameter, and featuring a 41 mm wall thickness, the component was manufactured in accordance with the American Petroleum Institute’s (API) rigorous API 20S standard and met all test acceptance criteria. Additionally, the component successfully passed industry-standard high-pressure tests as well as acceptance tests compliant with the widely used American ASME B31.3 standard. Figure 6 shows the pipe during the construction cycle.

Pressure testing was conducted by Trushape Engineering, a company specializing in high-pressure pipeline testing. The component was subjected to a pressure of 35 MPa for 60 min, maintaining its structural integrity throughout the process. The tests were supervised by the independent classification society Lloyd’s Register.

**Figure 7:**
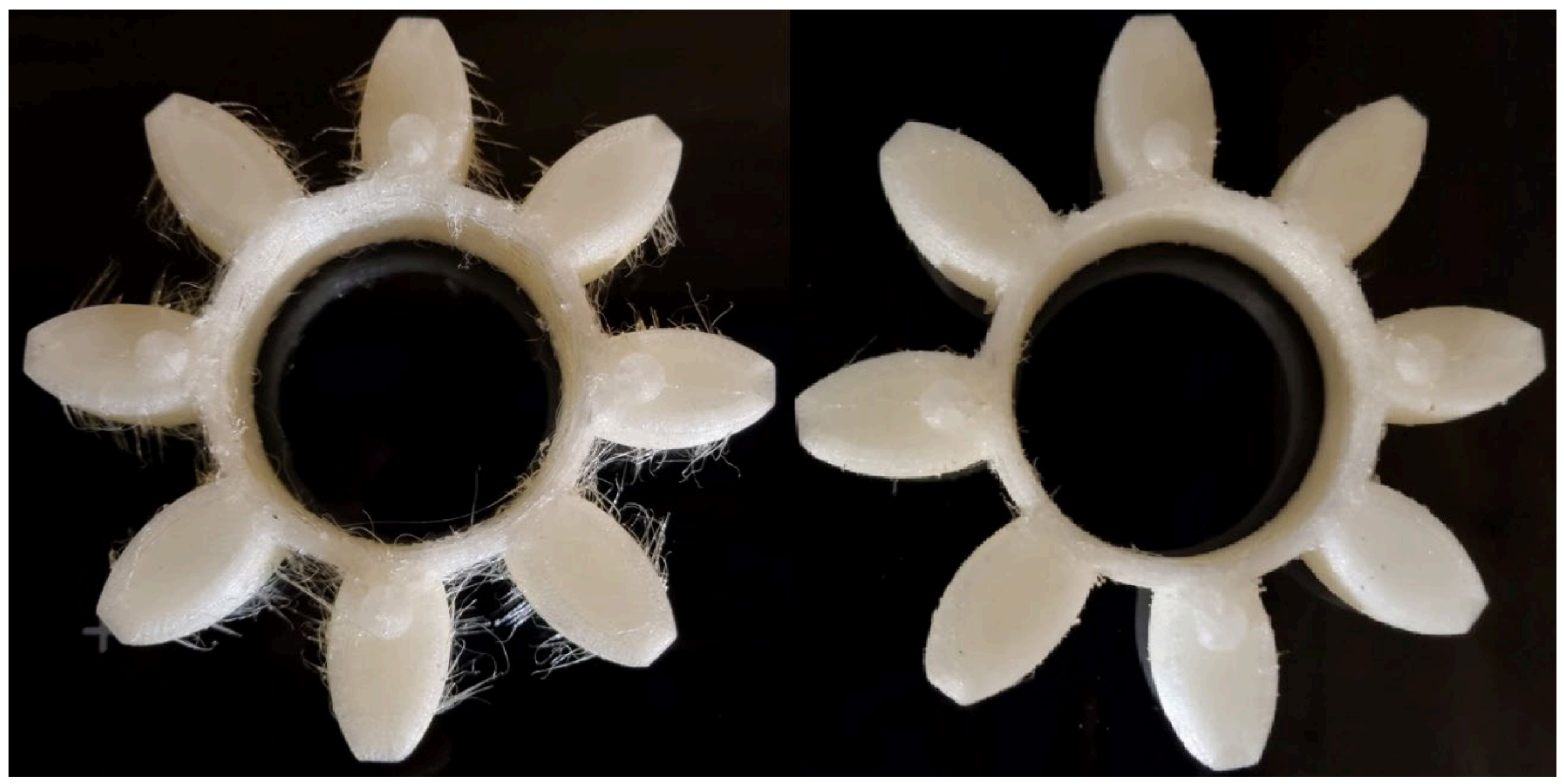
Flexible insert for a jaw coupling: as-printed (**left**) and after mechanical cleaning (**right**).

The paper also includes case studies based on our own work. This case study is a flexible insert for a jaw clutch (Section 3.3). Further on, the paper describes the individual components of the piston–connecting rod assembly: the piston, connecting rod, piston pin, bolt, and nut (Section 3.4). The wide range of materials available for additive manufacturing processes also enables the production of components made from synthetic polymers; processing these materials is generally relatively fast and does not require significant investment in machinery. The production of polymer components employs dedicated 3D printers utilizing the material extrusion method. The flexible insert for the ROTEX^®^ 42 jaw coupling is shown in Figure 7, manufactured using an HBOT 3D F300 printer. Parts for use in mechanical engineering that can be easily manufactured using 3D printing were proposed. These parts can be manufactured using any additive technology; in the presented cases, this technology was chosen due to its availability and low production cost. The manufactured elements were not designed to carry loads; therefore, thermoplastic materials used in 3D printing will work very well in these applications. ME printers are simple to use, take up little space, and are less sensitive to environmental conditions than more advanced technologies. This is the most likely role for 3D printing onboard ships, given the current state of technology and solutions used in shipbuilding. In specific cases, more advanced methods can be used, after preparing the infrastructure and crew, but this requires research into which events and scenarios would justify the cost and effort of implementing 3D printing onboard so that the benefits outweigh them.

## 3. Results

### 3.1. Case Study of the WAAMpeller Propeller

Available technology, supported by both technical and economic considerations, allows for the production of marine propellers using three main methods:

- Subtractive machining from a large solid block of material;
- Casting;
- Additive manufacturing.

Material removal machining is characterized by widespread availability, low production setup costs, and relatively high quality and productivity metrics, all of which contribute to the common use of these methods in manufacturing metal and non-metal components. However, when applying this method to the production of marine propellers, additional factors must be considered: significant material loss, the inability to create complex geometric structures, and the inability to modify the material’s local chemical composition to meet specific technological requirements. A frequently used indicator of cost-effectiveness in machining processes is the BTF (Buy-to-Fly) ratio, which represents the relationship between the amount of material used in production and the actual weight of the finished part. A high BTF ratio indicates a substantial loss of input material during machining; this directly increases raw material costs and adds expenses and operations related to waste processing, making it highly desirable to keep the BTF ratio as low as possible. Producing components with complex geometries such as marine propellers using material removal methods typically results in high BTF ratios. In practice, if a high BTF ratio is projected for a component during the design phase, alternative manufacturing methods that minimize material waste should be sought.

Casting is a widely used method for manufacturing large components with complex geometries that would be economically or technologically unfeasible to produce via other available methods. Key advantages of this method include: minimal material waste (primarily from finishing operations); relatively low production costs for medium- and high-volume runs where casting molds and techniques can be employed to significantly reduce production preparation time and costs; and relatively few size limitations for the cast components. However, in the case of the one-off production of a ship propeller sized according to the WAAMpeller design, despite a low Buy-to-Fly (BTF) ratio favoring casting, the process entails numerous economic, technological, and environmental drawbacks. These include: the inability to influence the process while the casting is being formed; the fact that quality verification is only possible after casting is complete; limited ability to shape the component’s internal geometry; the inability to control local material properties or vary material composition in specific geometric areas; lower quality parameters (regarding geometric tolerances); high energy consumption; and a costly, time-consuming production preparation process with the latter factor having the greatest influence on the choice of manufacturing method.

Sand casting is the most common method used for the one-off production of large ship propellers. To achieve the desired product shape using CNC machine tools or a robotic machining cell, a pattern of the screw made of wood or EPS and incorporating casting allowances is created; subsequently, this pattern is used to construct the bottom and top sections of the sand mold, and the contact surface undergoes a firing process to prevent molten metal from penetrating the mold. Once firing is complete, the mold is assembled and filled with molten metal. The subsequent stages involve cooling the casting, dismantling and disposing of the mold, and performing final processing on the screw through grinding and polishing operations.

The entire process of preparing the pattern and casting mold is time-consuming and costly, requiring experience and precise control, as errors made at this stage can result in the rejection of the finished casting.

WAAM technology is an alternative method for manufacturing marine propellers; it utilizes a robotic additive manufacturing station adapted through the implementation of gas-shielded arc welding (using wire feedstock) and the integration of software and equipment to build the component layer by layer. The method is characterized by a very low Buy-to-Fly (BTF) ratio, relatively short production preparation and manufacturing times, and the ability to control technical process parameters. It offers extensive capabilities for shaping external and internal geometries, depositing different materials within the geometric structure, and locally controlling material properties, all while consuming less energy than the alternative methods described above. Applying the WAAM method to produce components from a NAB (Nickel–Aluminum–Bronze) alloy depending on the workpiece orientation within the build envelope can yield increases in tensile strength (50%), yield strength (20%), elongation (60%), and hardness (28%), as well as improved wear resistance compared to casting technology [26]. This method aligns well with Industry 4.0 concepts as a modern, eco-friendly, and economically viable approach to manufacturing high-performance components, enabling their design, production, and refurbishment while overcoming previous technological limitations. The greatest challenge in designing and monitoring the WAAM process is ensuring appropriate process parameters throughout the entire build cycle; this is particularly critical because, in layered manufacturing, defects in a lower layer can affect every subsequent layer. Achieving this requires proper integration between the software and the manufacturing hardware. Production planning must also take into account the anisotropic nature of the component produced via the WAAM process.

A case study of the world’s first ship propeller to receive classification society certification demonstrates the significant potential of additive manufacturing methods for marine components specifically the WAAM process. This method enables superior material efficiency, thereby improving cost-effectiveness and reducing the environmental footprint compared to conventional techniques, while simultaneously enhancing the component’s technical properties. Furthermore, the use of wire feedstock significantly lowers production costs relative to powder-based additive manufacturing methods and boosts production efficiency.

### 3.2. Case Study of a DN400/DN300 Schedule 160 Pipe

**Figure 8:**
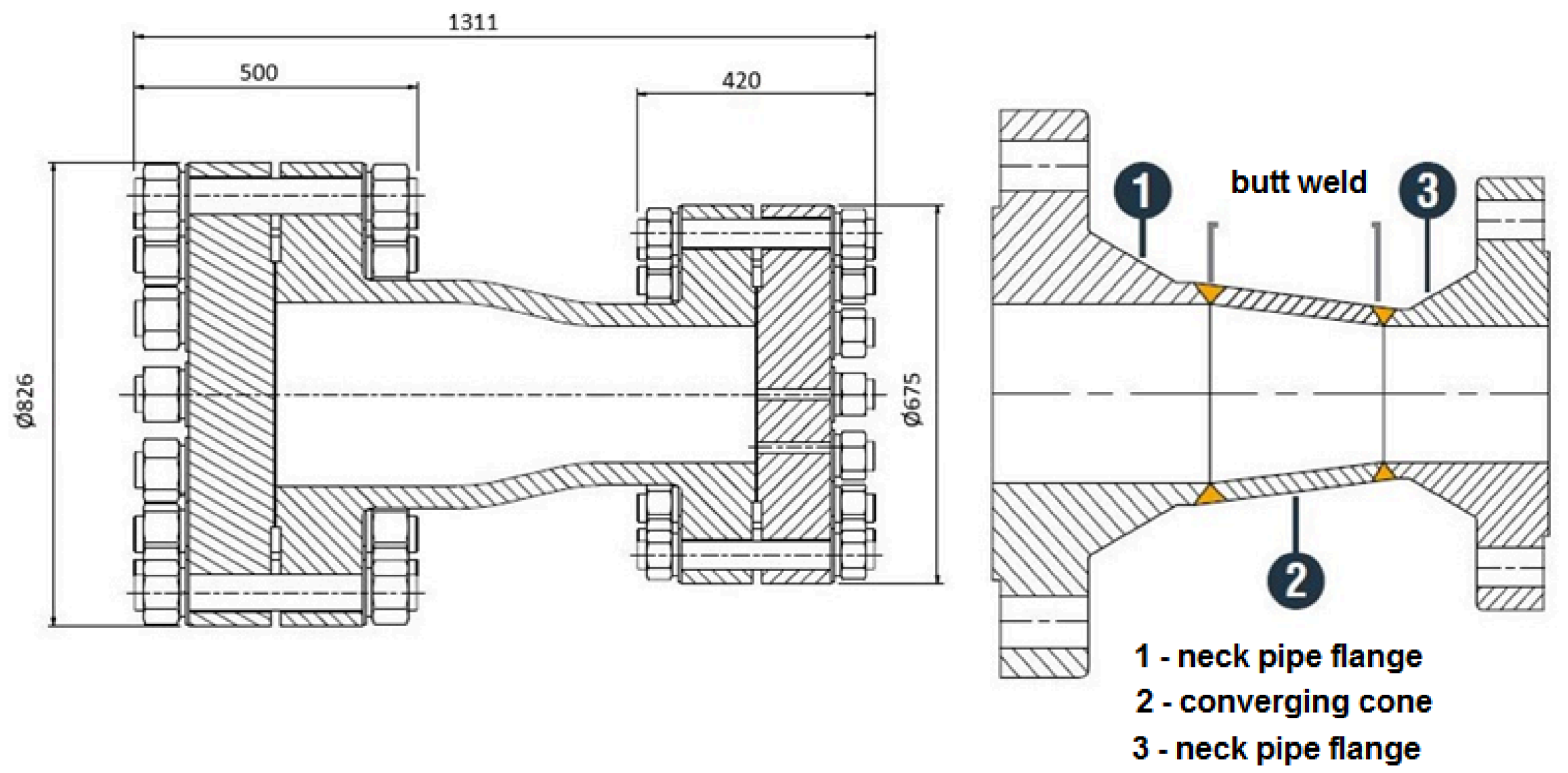
Comparison of product manufacturing: using the WAM^®^ method (**left**) versus welding prefabricated components (**right**) [25].

Available technology, supported by both technical and economic considerations, enables the production of a pipeline reduction component using two main methods: welding the pipeline’s constituent parts or employing additive manufacturing. Figure 8 presents a comparison of the product resulting from the use of each method. The 940 kg component is a high-pressure piping spool, 850 mm in length, 450 mm in diameter and 41 mm thick. The spool was additively manufactured in one piece using AML3D’s Wire Additive Manufacturing (WAM^®^), also known as Wire Arc Additive Manufacturing (WAAM), a wire-based form of Directed Energy Deposition (DED).

Manufacturing via welding is the most common method for manufacturing pipeline components and involves joining prefabricated elements to create a product that meets the specific technological and geometric parameters required for a given installation. This method is widely used primarily due to the relatively low cost of producing components with diverse geometries, the efficiency of the manufacturing process, extensive experience in production, certification, and acceptance testing, the availability of prefabricated parts, and the lack of a need for finishing machining.

However, achieving a component with specific dimensions even in the case of relatively small pipeline parts requires welding to join the constituent elements. While using available prefabricated components is economically justified as it significantly reduces production lead times and tangibly improves process profitability, it also limits manufacturing options to a specific inventory of parts with predefined geometries and chemical compositions. Achieving a specific geometry tailored to a custom design requires casting or hot forging, processes that entail time-consuming and costly preparations for small-batch production. Preparing pipeline components for welding involves readying the parts in accordance with welding procedures and performing a preliminary assembly of the prefabricated elements; this stage directly impacts final quality, and any errors made during this phase determine the serviceability of the finished component. Common installation errors include, for example, failure to maintain parallelism between flange mating surfaces, incorrect flange alignment relative to the pipeline, misalignment of flange bolt holes, improper preparation of surfaces for welding, incorrect execution of the welding process, and incorrect pipeline length or geometry.

Manufacturing using the WAM^®^ technology carried out by AML3D demonstrates an alternative method for manufacturing pipeline components using the additive manufacturing process. This method employs a robotic welding station integrated with a rotary welding table, along with auxiliary equipment such as a gas preparation unit. Before production begins, it is necessary to create a digital model of the component, select the manufacturing path and process parameters, and perform calibration and setup of the welding robot. The pipeline component produced via the WAM^®^ process must subsequently undergo subtractive machining to achieve the required quality specifications. In the case of the DN400/DN300 Schedule 160 pipe manufactured by AML3D, 1120 kg of material was deposited during the build process, while 180 kg of excess material was removed during post-processing using subtractive techniques.

The WAM^®^ method employed by AML3D offers an alternative to conventional manufacturing processes; data derived from a case study of a manufactured component benchmarked against standard techniques demonstrate that it significantly reduces delivery time. Specifically, the time required drops from over 10 weeks (when producing the final part from internationally sourced preforms) or over 3 weeks (using locally supplied preforms) to approximately 1.5 weeks with the wire additive manufacturing, a time frame comprising a 216 h build cycle, 12 h of subtractive machining, and the remainder allocated to intermediate production stages [10,25].

### 3.3. Flexible Insert for a Jaw Coupling

The wide range of materials available for additive manufacturing processes also enables the production of components made from synthetic polymers; their processing is typically relatively fast and does not require significant investment in machinery. The production of these polymer components utilizes dedicated 3D printers employing the ME method.

**Figure 9:**
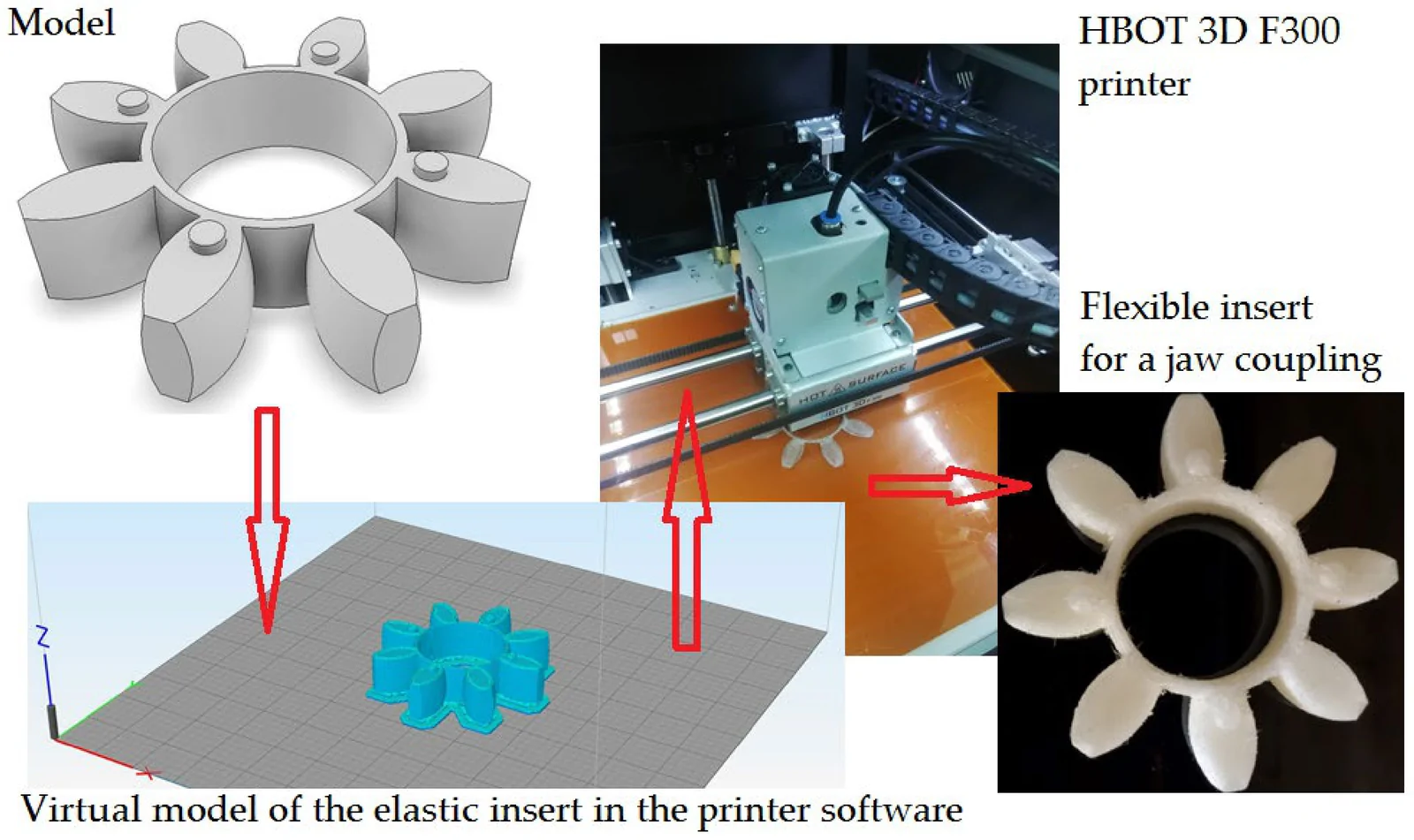
The process of 3D printing a flexible insert using the ME method.

The insert was printed in response to a need arising from damage to an original part within a safety-critical fire protection system on a vessel (a passenger and car ferry). After diagnosing damage to the flexible element of the pump assembly, the entire jaw coupling was inspected; the results showed no damage to the aluminum components, allowing for maintenance to be limited to replacing the flexible coupling. The preferred repair method is to replace the damaged part with a new one, specifically adapted to the application and recommended by the fire protection system manufacturer. However, situations involving limited availability (such as a breakdown during transit, long delivery times to port, or a complete lack of parts at the distributor) make 3D printing a viable alternative for obtaining the part necessary to maintain system safety. The production process for the flexible insert followed the workflow illustrated in Figure 9.

The component fabrication phase within the printer accounted for the largest share of the total process time, representing 92%. It is worth noting that the digital model preparation stage could be entirely bypassed, as the model for the required part was available in the manufacturer’s digital parts library. Establishing the technological parameters for the part to be printed is a critical stage in the ME-based manufacturing process; omitting or incorrectly specifying key input data can significantly affect the manufactured part’s suitability for its intended application. During the parameter-setting stage, the type of damaged component was defined, along with the parameters listed in Table 1. These parameters were selected to ensure the most accurate possible reproduction of the original product’s features.

Table 1 lists typical values for this type of TPU (thermoplastic polyurethane) material. To verify hardness, hardness measurements were performed using a Type D durometer and consistency with the hardness specified in the manufacturer’s documentation was determined.

**Figure 10:**
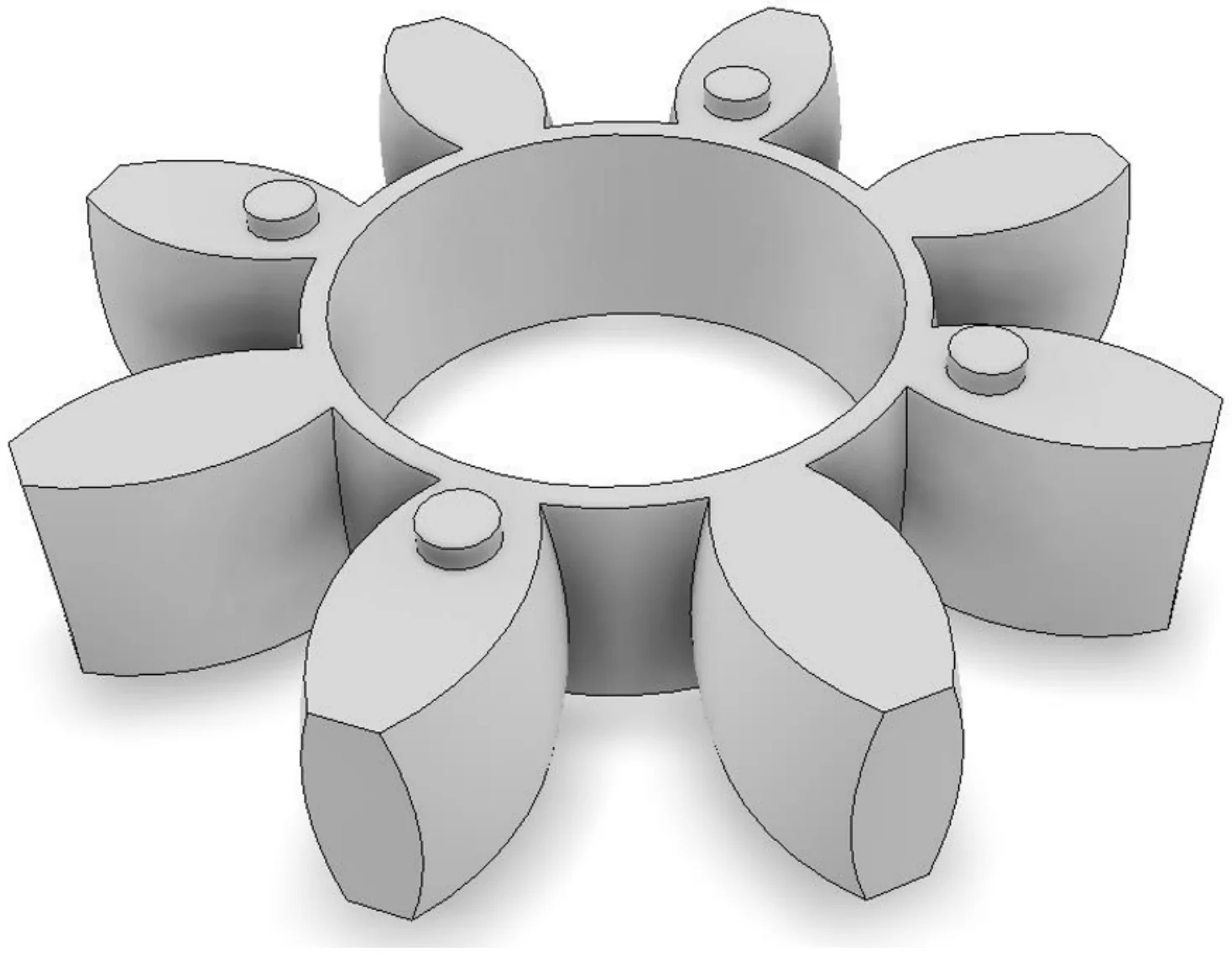
Virtual model of an elastic insert rendered in the CAD environment.

Downloading the virtual part model. The virtual model of the component shown in Figure 10 was obtained from a digital catalog provided by the part manufacturer in .stp format.

Transfer of the model to the 3D printer software. The digital model, provided by the part manufacturer in .stp format, was uploaded to the printer software for further processing, orientation within the build space, and generation of the command sequence.

**Figure 11:**
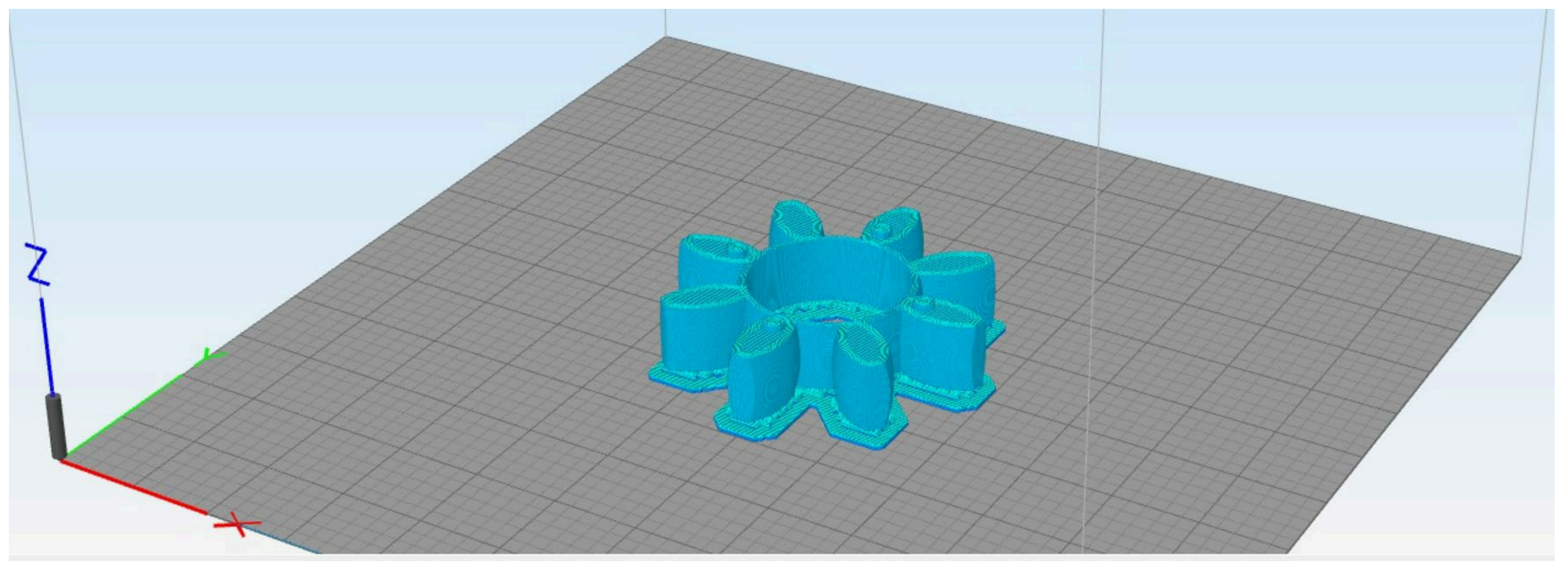
Virtual model of the elastic insert in the printer software environment.

The model imported into the printer software environment is shown in Figure 11.

The print parameters were determined after transferring the digital model to the printing device’s operating environment, taking into account input parameters as well as the recommendations of the filament and printer manufacturers. The print parameters set by the operator are presented in Table 2.

Other print parameters, including extruder settings, layers, infill, supports, temperatures, cooling, G-code, speeds, additives and other configurable settings, were automatically determined by the printer software based on parameters entered by the operator. Generating and transmitting the G-code constitutes the final step before the manufacturing cycle begins. The 3D printer uses the same command language as the numerical control machine tools employed in other manufacturing processes. Once all print parameters were established, a G-code file containing instructions for the build process was generated. This file was transferred directly to the printing device. A snippet of the G-code, containing commands corresponding to the specified parameters and the start of the print path, is presented in Appendix A. The build cycle commenced following the successful verification of all input parameters, acceptance of the part’s orientation within the machine’s build volume, and the successful transfer of the G-code file. The printer software estimated the build cycle duration at 7 h and 52 min; however, the actual process took 7 h and 12 min. Post-processing involves the mechanical removal of excess material formed during the nozzle’s movement between the contour surfaces of the component being built. Quality control is the last element of the production cycle and consists of verifying the quality parameters of the manufactured product, including assessing the suitability of the manufactured part for use in the pump system drivetrain assembly. The control of a 3D printing component is typically established according to the following procedural hierarchy: Quality Management System (facility-level procedures already covered by relevant standards); facility procedures—describe what activities are performed, who performs the activities, and when; work instructions—(or standard operating procedures) describe how activities are performed and provide more detailed instructions; and records—record specific details during the execution of activities related to the work instructions.

After diagnosing damage to the flexible element in the pump assembly, the entire claw coupling was inspected; the results obtained did not indicate damage to the aluminum components, which allowed for limiting the service work to replacing the flexible coupling. In the event of damage, the preferred repair method is to replace the damaged element with a new one, adapted to the task at hand and recommended by the fire protection system manufacturer. However, in cases of limited component availability, such as a failure during a voyage, extended delivery time to port, or complete unavailability of the part from the distributor, an alternative method for obtaining the part required to maintain system safety is to manufacture it using additive manufacturing methods. After installing the 3D-printed element in the pump, the correct operation of the entire assembly was determined. Employing the ME method in the aforementioned application enabled the relatively rapid acquisition of a part essential for restoring the operational readiness of a critical system on the vessel. The primary factor determining the use of additive manufacturing technology for this component was the time required to obtain the spare part. For systems critical to vessel safety, it is essential to ensure the shortest possible repair time and a rapid response to maintenance needs, thereby minimizing the duration of reduced operational readiness or technical performance. In practice, the primary factor extending the time between system failure detection and the initiation of corrective actions is often the limited availability of required parts from local suppliers; this necessitates waiting for delivery from the manufacturer’s representative or a central distribution hub and entails logistical challenges, such as determining the vessel’s location in time for the scheduled delivery. Utilizing additive manufacturing (AM) to produce parts just in time either directly at the facility requiring the part or at the port where the vessel is currently docked allows for a significant shortening of the supply chain. This directly minimizes repair time, thereby enhancing the safety of the crew, passengers, property, and the vessel itself.

### 3.4. Design of a Piston and Connecting Rod Model on a 3D Printer

The project aimed to design a piston and connecting rod assembly for an internal combustion engine and subsequently print it using 3D printing and the Material Extrusion (ME) method. The design was based on the actual configuration used in the first-generation Chevrolet V8 5.7L/350 automotive engine, produced between 1969 and 1990. As this is a conceptual design intended to demonstrate the principles of 3D printing, specific dimensional details for the individual components have been omitted.

**Figure 12:**
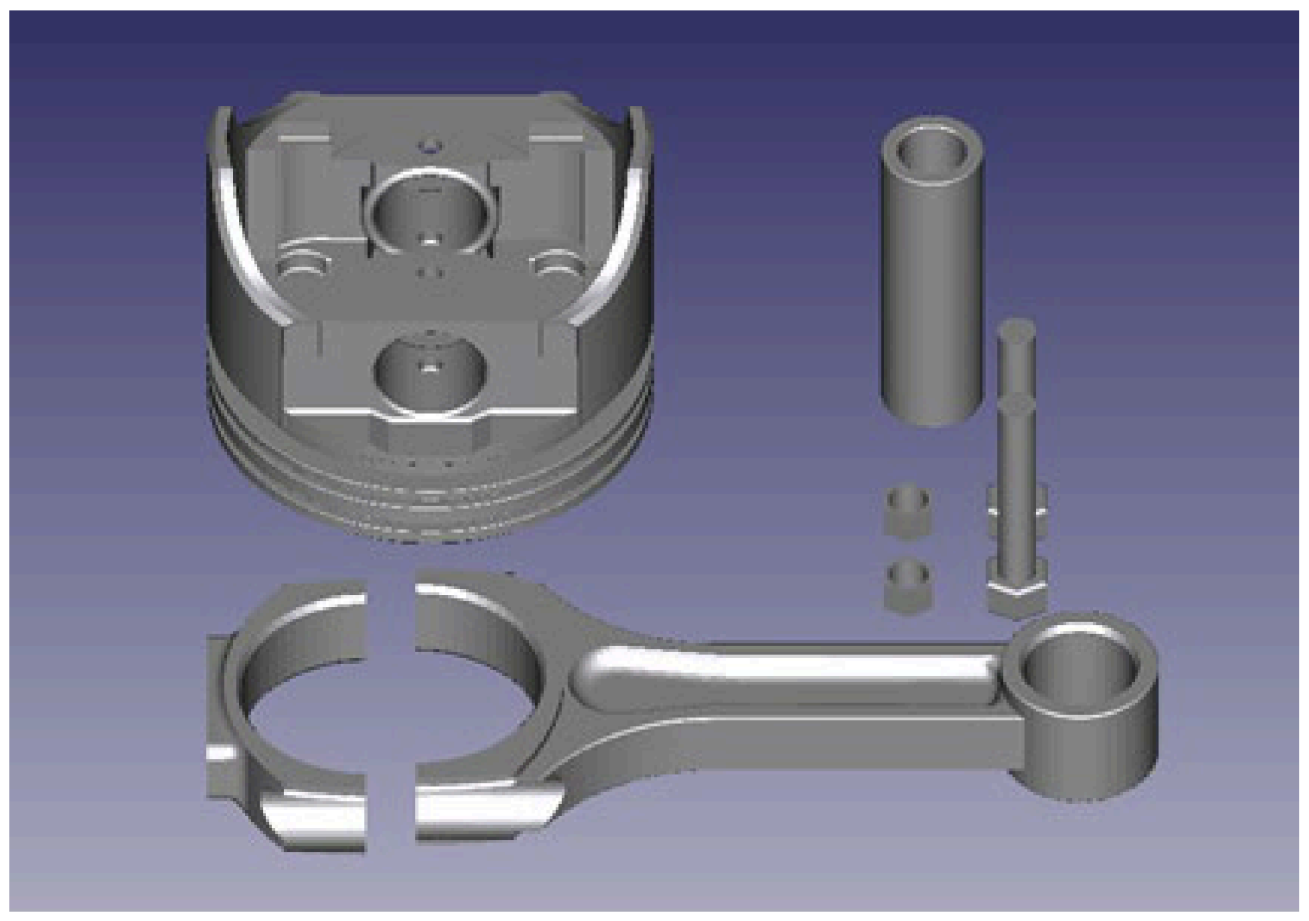
The design components and their representations—piston, connecting rod, gudgeon pin, bolt, and nut.

The design phase involved creating a model using Computer-Aided Design (CAD) software. After modeling the individual components, each was separately converted to the *.stl format. Each designed part is presented alongside its representation as a geometric triangle mesh, the result of converting the 3D solid into the *.stl format. Figure 12 shows the complete set of designed parts (piston diameter nominal: 100 mm).

The 3DGence One Slicer printer was used for printing, the technical specifications of which are presented in Table 3.

Dedicated software 3DGence One Slicer was used to configure the print parameters. The installed nozzle had a diameter of 0.4 mm, and the filament used was polylactide (PLA) with a diameter of 1.75 mm and a printing temperature range of 190–265 °C. The extruder temperature was set to 265 °C and the build plate temperature to 60 °C.

Previously generated *.stl files were loaded into the software. Before printing, it was necessary to define the layer height and infill density for each component, as well as to enable the automatic generation of support structures. Once these parameters were set, the “slice” function was used to automatically divide each component into individual layers and generate supports for protruding and overhanging sections. Details regarding this process and the estimated printing time for each component are presented in Table 4. The final step involved generating a file containing printer commands (G-code) for each component. These files were saved to an SD memory card, which was then inserted into the printer’s card slot. Each component was printed individually.

In Table 4, different layer heights and infill densities were selected for the components. The main observation regarding the project is the long overall printing time characteristic of the printing method used. This time depends primarily on the print settings (including single-layer height, infill level, use of support structures, and orientation of the object on the build plate) and the properties of the printed part (size and complexity). Reducing the single-layer height and increasing the infill level can increase the printing time several times, although this results in improved accuracy and durability. The way the printed element is oriented on the build plate before printing has a significant impact on the printing time. This determines the arrangement of support structures (if automatically generated by the program), if they are even necessary. Each additional structure increases the amount of filament required (which will effectively become waste), resulting in an increase in the overall printing time. Removing support structures during finishing can carry the risk of damaging the workpiece surface at the contact points, resulting in a deterioration of its aesthetic qualities. These structures are generally easy to remove manually, although difficulties may arise in hard-to-reach areas, narrow holes, and recesses. Therefore, it would be optimal to orient the object on the build plate so that the contact points occur on visually insignificant surfaces. It should be noted that this approach may significantly increase the printing time.

After printing each component individually and removing them from the build platform, they underwent a three-stage finishing process. In the first stage, the base layer generated at the start of printing was removed, and the support structures were detached by snapping them off or cutting them away with an appropriate tool.

**Figure 13:**
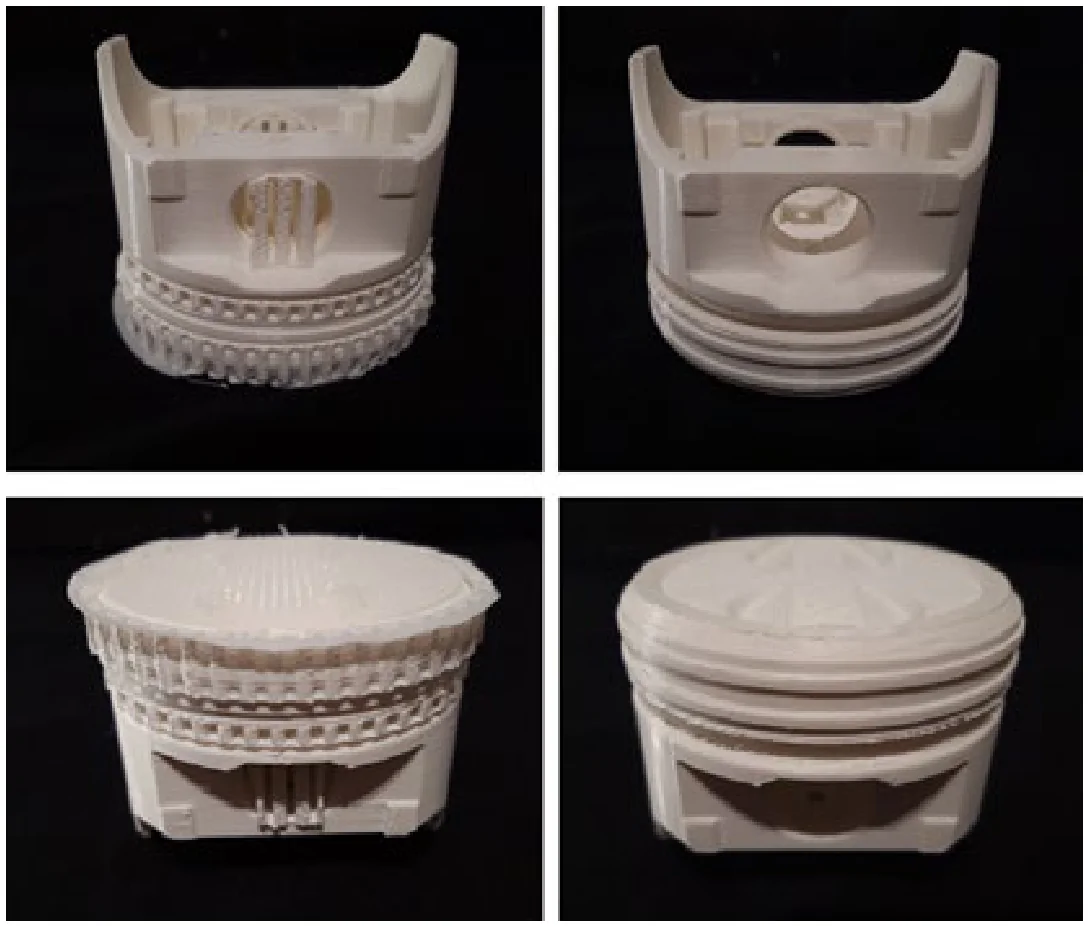
The piston before (**left**) and after (**right**) the removal of support structures.

**Figure 14:**
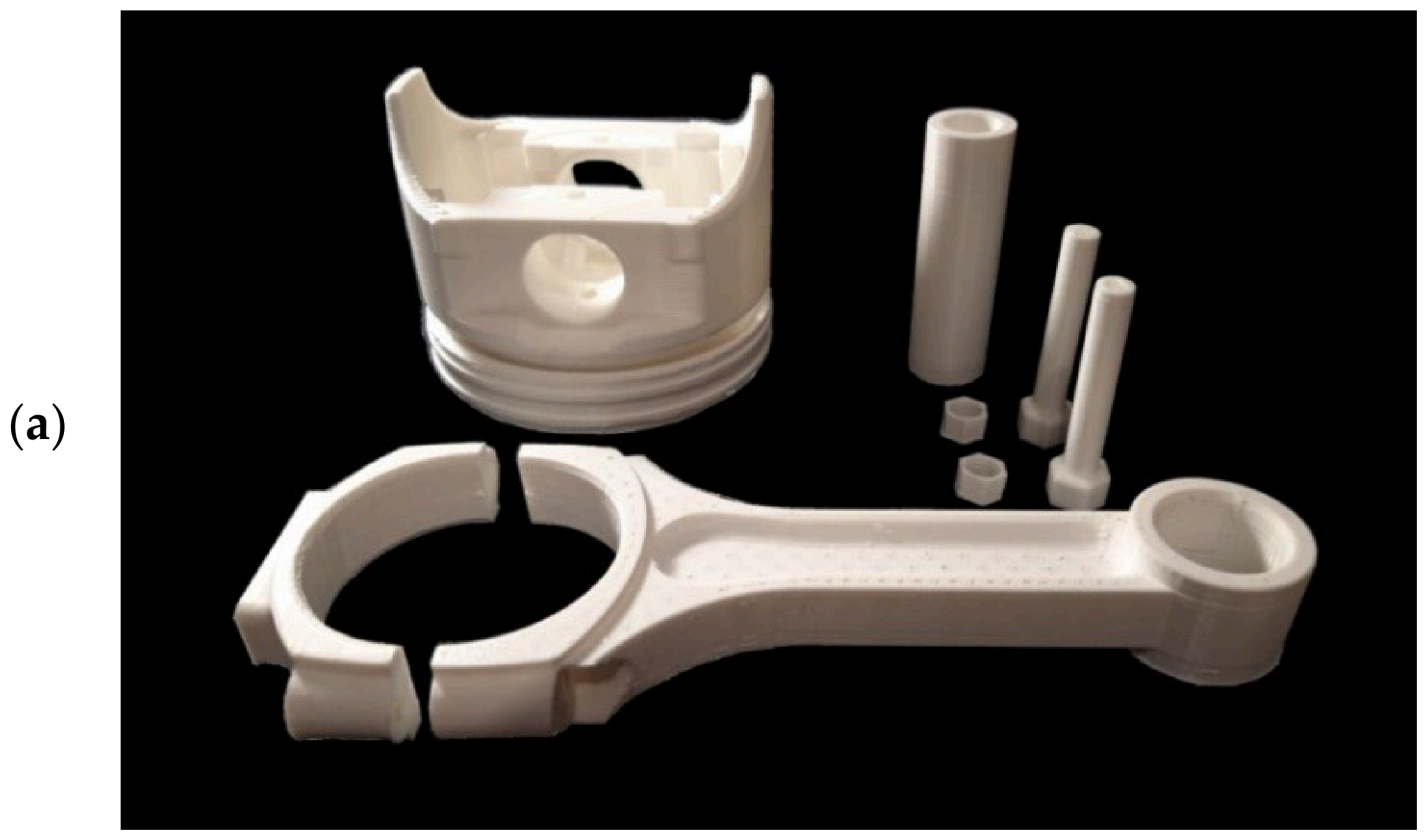

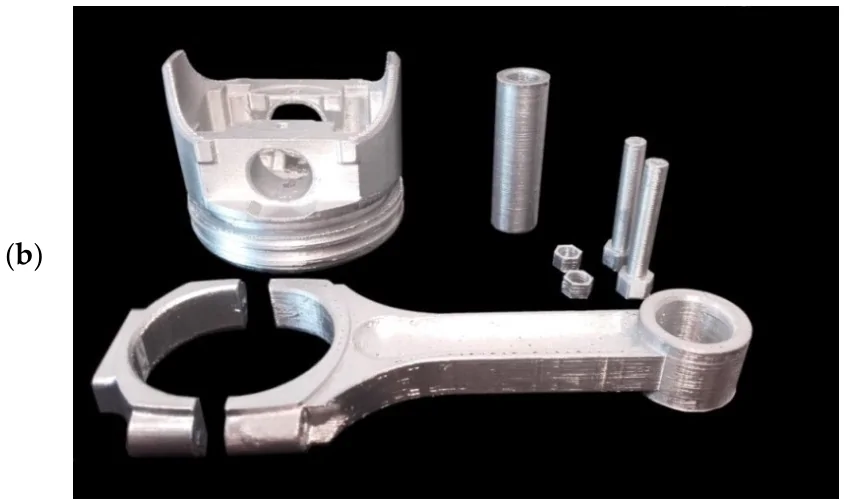
Models of printed components: (**a**) after removing excess material; (**b**) after painting.

**Figure 15:**
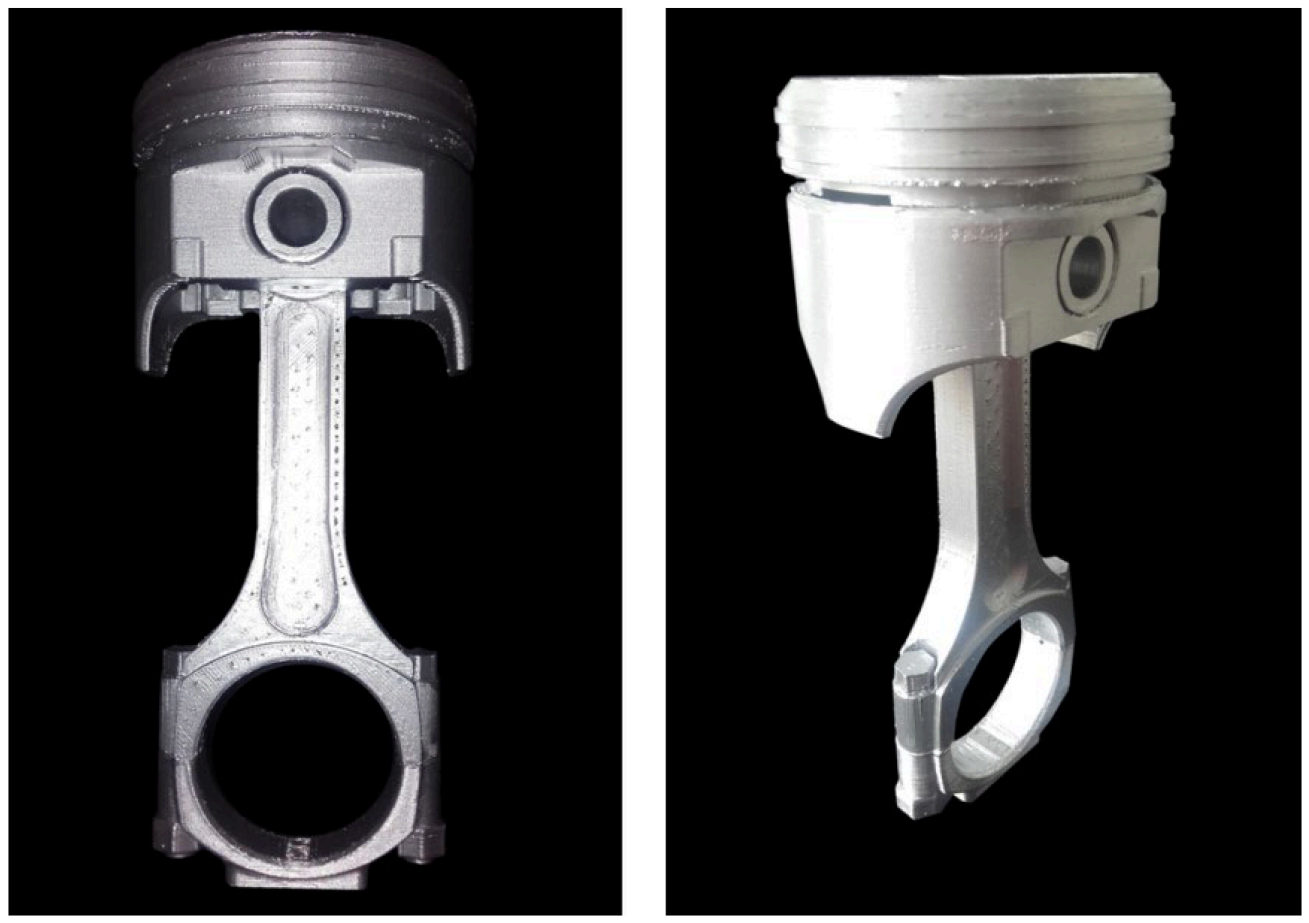
The finished model assembled into a piston–connecting rod unit: after painting (**left**) and after final finishing (**right**).

The finishing stage is illustrated in Figure 13, Figure 14 and Figure 15. The final step involved painting each component silver and allowing the assembly to dry for 24 h. Once dry, the unit was assembled, painted again, and left to dry for another 24 h. This is how the piston–connecting rod assembly model was prepared.

## 4. Discussion

In production, a number of tests are carried out on our products to estimate their durability, but this is based on exemplary operating conditions, which are often very difficult or impossible to maintain for 100% of their operating time. Therefore, periodic inspections are crucial, aimed at detecting and correcting minor defects before they lead to equipment downtime. These defects can result from material defects, improper machining, a lack of dimensional tolerances, assembly defects, poor operation, or wear. Therefore, it is crucial to regularly inspect all critical components, as not all defects are solely due to time and improper operation.

The operating and maintenance documentation for marine combustion engines distinguishes so-called wear parts, i.e., components whose lifespan is assumed to be shorter than the lifespan of the entire engine. Wear parts must be renewed during the engine’s operation. Renewal may involve replacing a damaged component with a new one, replacing it with a repaired component, or repairing the component during an overhaul. Only components whose service life has not been exhausted and repair costs are significantly lower than the price of a new component may be repaired. In the case of marine engine components, the first condition may be met if the component’s damage was caused by tribological, erosive, or corrosive wear.

Due to technological (technical parameters, physicochemical and mechanical properties), quality-related, and economic considerations, the use of additive manufacturing methods in the production of components for the shipbuilding industry as well as other sectors enables the creation of products that would be impossible to obtain using conventional manufacturing techniques. Furthermore, beyond technological and economic factors, the adoption of additive methods is justified by characteristics inherent to these processes, including: a reduced environmental footprint resulting from minimized material waste and shortened supply chains; flexible production management through the relatively easy transfer of manufacturing to facilities with the necessary technological infrastructure and skilled workforce; the potential to reduce physical spare-part inventories by producing parts “on demand”; the ability to manufacture spare parts directly at the site where the technical asset is in operation; and time savings in the production and delivery of required products, particularly for parts that are scarce or no longer in production.

Numerous studies and publications indicate that additive methods are widely applied in both medium- and high-volume production of small-scale components primarily using metal powder-based processes and in the one-off or low-volume production of medium-sized (e.g., via WAM^®^ and WAAM) and large-scale components (e.g., via the BAAM method). A comparative analysis of additive manufacturing methods versus conventional techniques conducted on two distinct components produced using WAM^®^ and WAAM technologies with materials of differing chemical compositions, alongside a case study of a TPU component produced via the ME method, indicates that specific factors determine the suitability of additive methods for the component manufacturing stage.

When manufacturing a component such as a ship propeller, the key parameters determining the choice of additive manufacturing methods include

- Production preparation time;
- Manufacturing time;
- Post-processing time;
- The ability to monitor the production process in real time;
- The degree of geometric complexity;
- Material consumption (BTF ratio);
- Varying material requirements within the geometric structure;
- Energy consumption and the environmental footprint of the manufacturing process;
- The digitalization of production processes—particularly crucial for the production of individual components where the geometric shape and required technological parameters are derived from comprehensive computer analysis that accounts for both the operating environment and the interaction of individual components with the overall parameters of the designed vessel.

A case study on the production of a DN400/DN300 Schedule 160 pipeline component indicates that the key parameters determining the selection of additive manufacturing methods as the appropriate production approach include

- Production preparation time (including the time required to source components);
- Manufacturing time;
- Post-processing time;
- A reduction in the number of constituent parts (part consolidation);
- A range of materials available for the specific manufacturing method;
- External geometry/shape;
- Energy consumption and environmental footprint of the manufacturing process;
- Technical parameters, as well as the physicochemical and mechanical properties achieved through the applied manufacturing technology.

A case study of an elastic insert component reveals that the key parameters determining the choice of additive manufacturing methods for its production include

- Method availability (technology infrastructure and materials);
- Part acquisition time which directly impacted the unit’s restoration to full technical readiness and logistical constraints arising from the mobile nature of the asset requiring the part.

It is likely that the large-scale adoption of additive manufacturing technology in the maritime industry will entail transforming the component supply business model by shortening the logistics chain; this would involve shifting from centralized, large-scale physical warehouses to a trade in digital spare-part models intended for production at local, specialized additive manufacturing facilities, including production directly on-board ships and platforms. Equally likely is a transformation of the current certification model for individual additively manufactured components, as existing procedures are excessively costly and time-consuming, thereby hindering the industrial-scale application of these methods. One potential solution would be to implement a certification methodology based on model validation, process validation, probabilistic methods, and component conformity. Such an approach should encompass the entire product lifecycle from raw material certification and the intellectual property rights associated with the 3D model to the finished part, thereby enabling technology transfer through part production licensing.

The unit-based nature of the production of many components fosters the ongoing optimization of their design and manufacturing technology, leading to a gradual increase in the number of additive-manufactured components in new generations of marine vessels; this applies both to simple parts where a potential failure would have little impact on the vessel’s overall safety and to components critical for operational readiness, safety, and maneuverability, such as propellers. In the long term, a manufacturer of additive produced components possessing a digital database of certified parts will be able to manage production flexibly, including by transferring part of the manufacturing process directly to the end-user’s technical facilities.

## 5. Conclusions

Analyzing the literature and our own research, we can conclude that additive manufacturing methods are widely used in both medium- and high-volume production of small-sized components which applies primarily to methods using metal powder as a material and also in single- and small-volume production of medium- and large-scale components.

A comparative analysis of additive manufacturing methods versus conventional methods, conducted for two different components manufactured using WAM^®^ and WAAM technologies using materials with different chemical structures, as well as a case study of a component manufactured using the ME method, indicates that there are factors that determine the selection of additive manufacturing methods as the appropriate method for component manufacturing.

The analysis carried out in the article, along with the identification of technological, economic, and environmental parameters for producing parts through 3D printing, showed a positive assessment of the feasibility and appropriateness of using additive manufacturing to produce spare parts in the shipbuilding industry.

Before launching any new production run, it is essential to conduct an analysis to select the optimal method for the task. The choice of manufacturing method is influenced by a range of factors, technological, organizational, economic, and, increasingly, environmental, yet the overarching goal, regardless of the specific component being produced, is to optimize production processes to ensure the most effective use of available technology. Available manufacturing approaches can be categorized into conventional and alternative (modern) production methods, the latter being characterized by parameters that align well with current market needs.

Additive manufacturing methods which represent a breakthrough in terms of how the final product’s shape is achieved can certainly be classified as modern manufacturing techniques. Additive manufacturing is one of the so-called Industry 4.0 technologies capable of transforming the industry towards eco-efficient, high-performance, just-in-time component production while increasing the digitalization of the entire process.

## Figures and Tables

**Table 1:** Technological parameters established for the flexible insert.

Parameter	Unit	Value
Type and size	-	Connector 42
Dimensions	-	Compliant with the component manufacturer’s database model and technical documentation
Material	-	TPU (thermoplastic polyurethane)
Hardness	Sh D	64

**Table 2:** Print parameters set by the 3D printing process operator, an HBOT 3D F300 printer.

Parameter	Unit	Value
3D printing method	-	ME
Material type	-	TPU (thermoplastic polyurethane)
Nozzle type	-	F300 Nozzle 0.4 modified
Layer width	mm	0.42
Layer height	mm	0.20
Determining orientation within the workspace	-	Central setting
Maximum Printhead Temperature	°C	245
Maximum Printbed Temperature	°C	80
Operating Temperature	°C	15–32
Filling	%	100

**Table 3:** Technical specifications of the 3Dgence One Slicer printer.

Parameter	Unit	Value
3D printing method	-	ME
Build Space	mm	235 × 255 × 195
Build Platform	-	Heated ceramic plate
Minimum Layer Height	μm	50
Number of Printheads	-	One replaceable
Nozzle Diameter	mm	0.3–0.5
Filament Diameter	mm	1.75
Filament Types	-	PLA, ABS, thermoplastic materials
Maximum Printhead Temperature	°C	265
Maximum Printbed Temperature	°C	60
Operating Temperature	°C	15–32
Dimensions 3D printing	mm	375 × 380 × 470
Communication	-	SD card, USB
Power	W	220–600

**Table 4:** Layer thickness and infill for individual elements.

Element	Layer Thickness [mm]	Infill [%]	Estimated Print Time [hr:min]
Piston	0.35	10	6:01
Connecting rod shank	0.15	25	4:38
Connecting rod cap	0.25	20	1:26
Pin	0.15	25	1:35
Bolt	0.25	20	0:26
Nut	0.25	20	0:05

## Data Availability

The data presented in this study are available on request from the corresponding author.

## Glossary

ABS	acrylonitrile butadiene styrene thermoplastic polymer
AM	additive manufacturing
BAAM	big area additive manufacturing
CAD	Computer-Aided Design
EBM	electron beam melting
LENS	laser engineering net shaping
LC	laser cladding
ME	material extrusion
MIG/MAG	metal inert gas/metal active gas
PLA	polylactide
SLM	selective laser melting
SLS	selective laser sintering
TPU	thermoplastic polyurethane
TIG	tungsten inert gas
WAM^®^	Wire Additive Manufacturing
WAAM	Wire Arc Additive Manufacturing

## Appendix A

The rapid development of additive manufacturing technology over the past few years and the positive outlook for the coming years have led some classification societies to issue publications containing guidelines and recommendations for additive manufacturing methods, the application of which will enable final product certification. The roadmap for Shipyard 4.0 is shown in Table A1.

The intensive growth of interest in and dissemination of additive methods in manufacturing processes is mainly due to the development of technologies used in 3D printing processes and a greater availability of materials, as well as numerous advantages and design possibilities that cannot be achieved with traditional manufacturing methods. Frequent problems occurring in the shipbuilding supply chain, as well as the spread of 3D printing technology and the development of materials used in additive processes, are factors influencing the widespread use of these methods for the production of spare (and replacement) parts in the shipbuilding industry.

**Table A1:** Shipyard Digital Innovation Roadmap.

Shipyard 4.0		Year
**Real-time planning tools track progress & report problems**		**2025**
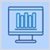
**Welding quality is digitally monitored in real-time**		**2025**
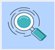
**AI-enhanced documents anticipate, forecast, and warn on events**		**2025**
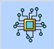
**Digital databases improve task training and handover**		**2026**
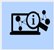
**Biocide-free coating innovations surpass toxic anti-fouling paints**		**2026**
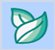
**AR, VR, and 3D simulation used in task training**		**2026**
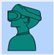
**AI systems control and manage warehouse inventory**		**2027**
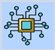
**3D Scanning is standardised for ship inspections**		**2027**
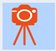
**Aerial drones perform incremental 3D scans at yards**		**2027**
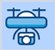
**Additive manufacturing of spare and missing parts**		**2028**
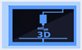
**Microgrids supplement yards’ electricity needs**		**2028**
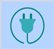
**Autonomous and flexible robots weld in hard-to-reach places**		**2028**
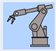
**Digital twin files are shared across stakeholders**		**2029**
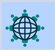
**Aerial drones perform basic services**		**2029**
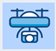
**Digital twins are used for virtual delivery inspections**		**2030**
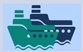
**Unmanned lifting vehicles displace tractor operators**		**2030**
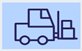
**Digital twins are used in planning repairs**		**2031**
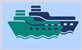
**Warehouse operations performed by automated drones**		**2031**
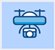
**3D printers are used to print large blocks**		**2032**
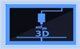
**Composite materials are used for large vessel hulls**		**2033**
